# Effect of Various Irrigation Rates on Growth and Root Development of Young Citrus Trees in High-Density Planting

**DOI:** 10.3390/plants9111462

**Published:** 2020-10-29

**Authors:** Said A. Hamido, Kelly T. Morgan

**Affiliations:** Southwest Florida Research and Education Center, University of Florida, Immokalee, FL 34142, USA; conserv@ufl.edu

**Keywords:** irrigation rate, planting density, canopy volume, trunk diameter, leaf area, root length, root longevity

## Abstract

Citrus yields have declined by almost 56% since Huanglongbing (HLB) was first found in Florida (2005). That reduction forced citrus growers to replant trees at much higher densities to counter-balance tree loss. The current project aims to determine how much water is required to grow citrus trees at higher planting densities without reducing their productivity. The study was initiated in November 2017 on eight-month-old sweet orange (*Citrus sinensis*) trees grafted on the ‘US-897′ (Cleopatra mandarin × Flying Dragon trifoliate orange) citrus rootstock planted in the University of Florida, Southwest Florida Research and Education Center (SWFREC) demonstration grove, in Immokalee, FL (lat. 26.42° N, long. 81.42° W). The soil in the grove is Immokalee fine sand (Sandy, siliceous, hyperthermic Arenic Alaquods). The demonstration grove included three densities on two rows of beds (447, 598, and 745 trees per ha) replicated four times each and three densities of three rows of beds (512, 717, 897 trees per ha) replicated six times. Each density treatment was irrigated at one of two irrigation rates (62% or 100%) during the first 15 months (2017–2019) then adjusted (2019–2020) to represent 26.5, 40.5, 53, and 81% based on recommended young citrus trees evapotranspiration (ETc). Tree growth measurements including trunk diameter, height, canopy volume, leaf area, and root development were evaluated. During the first year, reducing the irrigation rate from 100% to 62% ETc did not significantly reduce the young citrus tree growth. Conversely, the lower irrigation rate (62% ETc) had increased citrus tree’s leaf area, canopy volume and tree heights, root lifespan, and root length by 4, 9, 1, 2, and 24% compared with the higher irrigation rate (100%), respectively. Furthermore, the root lifespan was promoted by increasing planting density. For instance, the average root lifespan increased by 12% when planting density increased from 447 to 897 trees per ha, indicating that planting young trees much closer to each other enhanced the root’s longevity. However, when treatments were adjusted from April 2019 through June 2020, results changed. Increasing the irrigation rate from 26.5% to 81% ETc significantly enhanced the young citrus tree growth by increasing citrus tree’s canopy volume (four fold), tree heights (29%), root lifespan (86%), and root length (two fold), respectively. Thus, the application of 81% ET_c_ irrigation rate in commercial citrus groves is more efficient for trees from two to four years of age.

## 1. Introduction

Water scarcity is a critical dilemma in many areas around the world including Florida, and agriculture is the highest water consumption sector. During the 20th century, water demand increased twice the rate of population growth, by 2025, ≈66% of the world population is predicted to be located in countries under water stress conditions (500 ≤ capita ≤ 1000 m^3^) [1]. Besides, climate change intensifies this threat by causing frequent and severe drought events. While droughts can dramatically alter plant productivity and longevity, water excess can cause major damages such as wilting phenotype [2]. Diverse plant species manifest a contradictory wilting phenotype due to higher transpiration and lower root water uptake [2,3]. Thus, both water deficiency and excess negatively impact agriculture and result in significant economic losses.

In Florida, water is a limiting factor in citrus production because of the low water holding capacity and low organic matter content of Florida sandy soils. Increases in water use efficiency are accomplished by selecting the right irrigation scheduling program and application rate. Increasing water use efficiency would result in conserving water and reduce the loss of valuable nutrients from excess irrigation.

Various studies conducted in Florida during the last decade have led the University of Florida Institute of Food and Agricultural Sciences (UF/IFAS) to conclude that current citrus irrigation recommendations must be revised to appropriate amounts of water to improve tree canopy density. It has been concluded that once citrus trees are mature, they require approximately the same amount of water regardless of the in-row densities as long as the volume of the canopy is the same [4]. The best distribution of the root system is mainly limited to available water and nutrients in soil profile [5,6,7]. Citrus root system is anticipated to account for more than 60% of aboveground dry mass [8,9,10].

In addition, root diameter plays a significant role in soil penetration. Likewise, the average root diameter (ARD) is proportional to the root surface area which determined the extraction of nutrient and water. Thicker roots can penetrate harder soils [11,12]; therefore, better nutrient and water uptake than thinner roots. Penetrating soils and buckling stress are controlled by elastic and root diameters [13,14]. Bigger root diameters maintain greater buckling stress than smaller roots.

Leaves play a crucial role in providing roots with necessary carbohydrates, growth regulators, and organic synthesis [15]; thus, defoliation or reduction of leaf area may decrease or cease crop root growth. However, trees with lower leaf areas took up less water from the soil resulting in potentially thinner canopies [16]. The leaf area mainly impacts plant growth, photosynthesis rate, and productivity [17].

Florida citrus bearing acreage has been decreased by approximately 43% from 291 thousand ha of bearing trees in 2003 to 165 thousand ha in 2017 since Huanglongbing (HLB or citrus greening), a pandemic infection caused by the phloem-limited bacteria *Candidatus* Liberibacter asiaticus (*C*Lasiaticus) was first found in Florida in 2005 [18]. Citrus yields have also declined by almost 56% [18]. Reduction in yield from a state average of about 47.5 tons per ha in 2004 to 21.1 tons per ha in 2017 has forced citrus growers to replant trees at much higher densities. The rationale for higher density is anticipated trees loss from HLB and an attempt to increase yields in the early years (tree age 3 to 6 years).

Investigation of irrigation water application effects on young citrus trees at higher densities have not been documented in Florida. Thus, field-scale experimentation is performed in the current study to determine if current citrus irrigation practices need to be modified with different citrus trees densities. Therefore, the aim of the present study is to determine the amount of water required for young citrus trees at higher tree densities without a reduction in trees growth. Thus, the objectives were to determine the effect of irrigation amount on leaf area, canopy volume, tree heights, trunk diameter, and root growth and longevity of young citrus trees at selected densities.

## 2. Materials and Methods

### 2.1. Site Description and Experimental Setup

**Figure 1:**
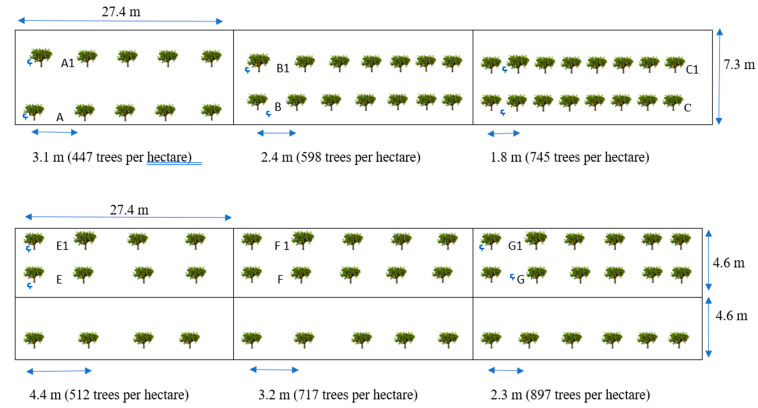
Diagram represents different planting spaces for two different replicates from two and three row beds with one emitter per tree with 53% ETc for A1, B1, E1, and F1 plots, one emitter per tree at 81% ETc for A, E, and F plots, one emitter per two tree providing 40.5%ETc per tree for B, C, and G plots, and one emitter per two trees providing 26.5% ETc per tree for C1 plot.

The experiment was initiated in November 2017 when eight-month-old “Valencia” (*Citrus sinensis*) trees grafted on the “US-897” (Cleopatra mandarin × Flying Dragon trifoliate orange) citrus rootstock were transplanted at the University of Florida, Southwest Florida Research and Education Center (SWFREC) experimental grove, in Immokalee, FL, USA (lat. 26.42° N, long. 81.42° W). The experimental area consisted of five-165-m-long beds with drainage swales on each side. Treatments consisted of three densities in two rows of beds (447, 598, and 745 trees per ha) replicated four times each, and three densities in three rows of beds (512, 717, and 897 trees per ha) replicated six times. Each density row treatment was irrigated at one of two irrigation rates (62% or 100%) based on recommended water use (ETc) for young citrus trees. Trees spacing were (A) 4.27 m apart in three rows, 4.57 m between rows (512 trees per ha), (B) 3.05 m apart in three rows, 4.57 m between rows (717 trees per ha), (C) 2.44 m between trees in three rows 4.57 m between rows (897 trees per ha), (D) 3.05 m apart in two rows 7.32 m between rows (447 trees per ha), (E) 2.29 m apart in two rows 7.32 m between rows (598 trees per ha), and (F) 1.83 m between trees in two rows 7.32 m between rows (745 trees per ha) (Figure 1).

Trees were irrigated by 360-degree micro-sprinklers (Maxijet Inc., Dundee, FL, USA) with one emitter per one tree or two trees at different discharge rates to meet the target irrigation treatments. The irrigation was separated into three irrigation systems, each supplying two plots (two replications) per bed (27.4 m long) of two or three rows. All trees were transplanted in Immokalee fine sand soil (Sandy, siliceous, hyperthermic Arenic Alaquods). Each plot is divided into two sub-plots during the first 15 months or four irrigation sub-plots during the last 17 months. Irrigation adjustments were due to higher moisture contents than field capacity for a higher irrigation rate during the first 15 months.

The initial two irrigation treatments provided 62% or, following the first 15 months, 100%, or four irrigation treatments provided 26.5%, 40.5%, 53%, or 81% of daily ET_c_ obtained from the Citrus Irrigation Scheduler found on the Florida Automated Weather Network (FAWN) website (http://fawn.ifas.ufl.edu/tools/irrigation/citrus/scheduler/) were examined. Weather data provided by the FAWN weather station, located within 100 m from the experimental site, is used to determine the amount of daily irrigation applied by a time clock every two weeks.

The adjustment in the irrigation rate favored maintaining the water content in the root zone near or below the optimum levels (80–90% of field capacity), thereby decreasing the total water rate.

Irrigation was supplied with a 0.172 MPa pressure pump to moisten a circular area of 10.2 m^2^ per tree (3.6 m diameter). Irrigation emitters were placed 33 cm away from the trunk at a single emitter per tree or in the middle between two trees at closer tree spaces.

The N and K were supplied bi-weekly by fertigation through separate fertigation lines delivering 0.036 m^3^ h^−1^, from mid-February to late-October, summing up 16 applications per year. The annual rate of N and K corresponded to 210 g tree^−1^ as ammonium nitrate and soluble potassium nitrate, respectively. In 2019, due to a higher precipitation rate than usual, fertilizer was not applied from 16-July through 26-August to reduce the risk of N leaching with taking into consideration to supply the mentioned fertilizer quantity during the fertigation cycles. The total precipitation from 16 July through 26 August was approximately 39% (464 mm) of the annual total rainfall of about 1178 mm. Therefore, that period introduced a high risk of N leaching.

### 2.2. Tree Growth Measurements

#### 2.2.1. Tree Height, Trunk Diameter, Canopy Volume, and Leaf Area

Tree height was measured during February and December of 2018, May and December of 2019, and May 2020 for all citrus trees. Tree height was measured from the soil surface to the highest point for each tree using a measuring stake. Trunk diameter was measured for all citrus trees in north-south and east-west directions using an electronic LCD digital caliper at a 30 cm height from the soil surface during February, May, and December 2018, May and December of 2019, and May 2020. Canopy volume was determined by measuring the vegetative canopy height and the average width of the canopy in two directions, east-west and north-south during December 2018, May and November 2019, and June 2020. The canopy volume (CV) was determined, as recommended by Obreza [19] in Equation (1):

CV = 0.5233 H W^2^ (1)

where CV is the canopy volume (m^3^), H is the height of the canopy (m), and W is the canopy depth measured in east-west and north-south directions (m). Leaves were counted on three to four trees per plot during May and December 2018. Average individual leaf area was estimated by collecting four to six representative leaves at different points within the canopy of each tree for a total of 20 leaves per plot. Individual leaf area was estimated using a portable leaf area meter (LI-3000A, LI-COR, Lincoln, NE, USA).

#### 2.2.2. Root Measurements

After transplanting, minirhizotron (MR) tubes were installed in the soil during 1 March 2018, using a 7.6 cm augar diameter for a total of 48 tubes. A 55 cm long acrylic MR tube (64 mm inner tube diameter) with a waterproof plug at the tube bottom was inserted 30 cm away from each tree trunk at 45°. Insulated caps were used to close the top of each tube to protect roots from light and heat stress. The total length of each buried tube was 40 cm. Twenty-seven days after tube installation (March 28), live root images were obtained every four weeks using a CI-600 In-Situ Root Imager (CID-Bioscience, Camas, WA, USA). Total roots length (TRL), the number of roots (NOR), and average root diameter (ARD) were determined. Live root images were analyzed using Root Snap CI-690 software (version 1.3.2.25, CID-Bioscience, Camas, WA, USA). Before measurements, the scanner was calibrated as needed using a manufacturer calibration procedure.

The lifespan of roots was also determined by tracking three to five individual roots per tree. Each root was tracked from the time of initiation to death (decay) at four weeks intervals.

### 2.3. Experimental Design and Statistical Analysis

This study was conducted as an irrigation treatment (2 or 4) × 6 tree densities (447, 512, 598, 717, 745, and 897 trees ha^−1^) factorial, complete randomized block design. The experiment included four replications of 447, 598, and 745 trees ha^−1^ and six replications of 512, 717, and 897 trees ha^−1^. Data were examined at Probability (*p*) ≤ 0.05 limits using a proper Statistical Analysis System for Windows (Software 9.4, SAS Institute Inc., Cary, NC, USA). In addition, root measurements, including TRL, NOR, and ARD, were analyzed over time as a repeated measure, and root longevity was analyzed using the Kaplan–Meier survival model [20].

## 3. Results and Discussion

### 3.1. Tree Growth Measurements

#### 3.1.1. Tree Height

From planting through the end of 2018, the two-way interaction irrigation rate × planting density (*p* = 0.1983) and irrigation rate alone (*p* = 0.6688) did not significantly affect young citrus tree heights. In the first seven months, an increase in height was very similar for all irrigation treatments. Similar observations were reported by Alves et al. [21]. In contrast, planting density alone statistically (*p* = 0.0094) influenced tree heights. Increasing planting density from 512 to 745 trees per ha significantly increased tree height by ≈60%. Trees growth was vigorous and reached 1.15, 1.06, 1.18, 1.20, 1.24 and 1.14 m heights by December 2018 for 447, 512, 598, 717, 745 and 897 trees per ha, respectively (Table 1).

When treatments were adjusted from May 2019 through June 2020, a different trend was observed. The two-way interaction of irrigation rate × planting density (*p* = 0.0600) and planting density alone (*p* = 0.4487) did not significantly affect the young citrus tree heights (Table 2). In contrast, irrigation rate alone statistically (*p* = 0.0245) affected tree heights [21]. Increasing the irrigation rate significantly increased tree height on average by 7% and 29% under 26.5% and 81% ET_c_, respectively (Table 2). Total increase under 81% ET_c_ was more than fourfold compared with 26.5% ET_c_ with an average daily height increase to be 0.02 and 0.09 cm under 26.5% and 81% ET_c_ irrigation rates, respectively.

#### 3.1.2. Trunk Diameter

In December 2018, the two-way interaction irrigation × planting density (*p* = 0.8494), planting density alone (*p* = 0.5058), and irrigation rate (*p* = 0.0527) did not significantly impact young citrus tree trunk diameter (Table 1) [21]. The average daily trunk diameter increase under 62% and 100% irrigation rate was 0.042 and 0.043 mm d^−1^, respectively.

When treatments were adjusted from May 2019 through June 2020, the two-way interaction irrigation × planting density (*p* = 0.0577) and planting density alone (*p* = 0.6195) did not significantly impact the young citrus tree trunk diameter (Table 2). However, the higher irrigation rate (81% ET_c_) significantly (*p* = 0.0282) increased tree trunk diameter of citrus trees on average by 29% compared with the lower irrigation rate (26.5% ET_c_). The average daily trunk diameter increase under 26.5% and 81% irrigation rate was 0.03 and 0.04 mm d^−1^, respectively. Reduction under lower irrigation rates was illustrated by Hsiao [22], who reported that reduced irrigation rate may reduce cell elongation in the trunk and rise with rising turgor potential. Turgor potential typically decreases with declining water availability. 

#### 3.1.3. Leaf Area

Although the leaf areas of all trees in the current project were similar at transplanting, significant changes occurred with time. Neither irrigation rate × planting density interaction (*p* = 0.9200) nor irrigation rate alone (*p* = 0.6341) significantly affected the young citrus tree leaf area at the end of 2018 (Table 1). Conversely, increasing planting densities from 447 to 897 trees per ha significantly (0.0029) increased young citrus trees leaf areas by ≈ twofold. Consequently, the average daily increase in leaf area was expected to be 60 and 110 cm^2^ d^−1^ under 447 and 897 trees per ha, respectively. The higher value is similar to leaf area increase reported for healthy trees by Hamido et al. [5]. The average monthly leaf area increase was from 0.18 to 0.34 m^2^ under 447 and 897 trees per ha, respectively. These data elucidate the impact of higher planting density on leaf area.

#### 3.1.4. Canopy Volume

At the end of 2018, when trees got bigger and shaded the ground, we started to collect the canopy volume. At December 2018, although planting density (*p* = 0.3888) and irrigation rate (*p* = 0.1564) did not significantly affect young citrus tree canopy volume, decreasing the irrigation rate from 100% to 62% ETc and increasing planting density from 447 to 897 trees per ha increased the canopy volume by 9% and 5%, respectively (Table 1).

When treatments were adjusted from May 2019 through August 2020, the two-way interaction irrigation × planting density (*p* ˂ 0.0001) significantly affected young citrus tree canopy volumes (Table 2). Increasing the irrigation rate to 81% ET_c_ increased tree’s canopy volume by ≈ threefold compared to the lower irrigation rate of 26.5% ET_c_. The average daily canopy volume increase was estimated to be 0.006 and 0.002 m^3^ d^−1^ under 81% and 26.5% ET_c_ irrigation rates, respectively. In contrast, increasing planting density from 447 to 897 trees per ha resulted in the canopy volume reduction of 17% with estimated canopy growth between 0.005 and 0.004 m^3^ d^−1^ under 447 and 897 trees per ha, respectively.

#### 3.1.5. Total Root Length and Number of Roots

**Figure 2:**
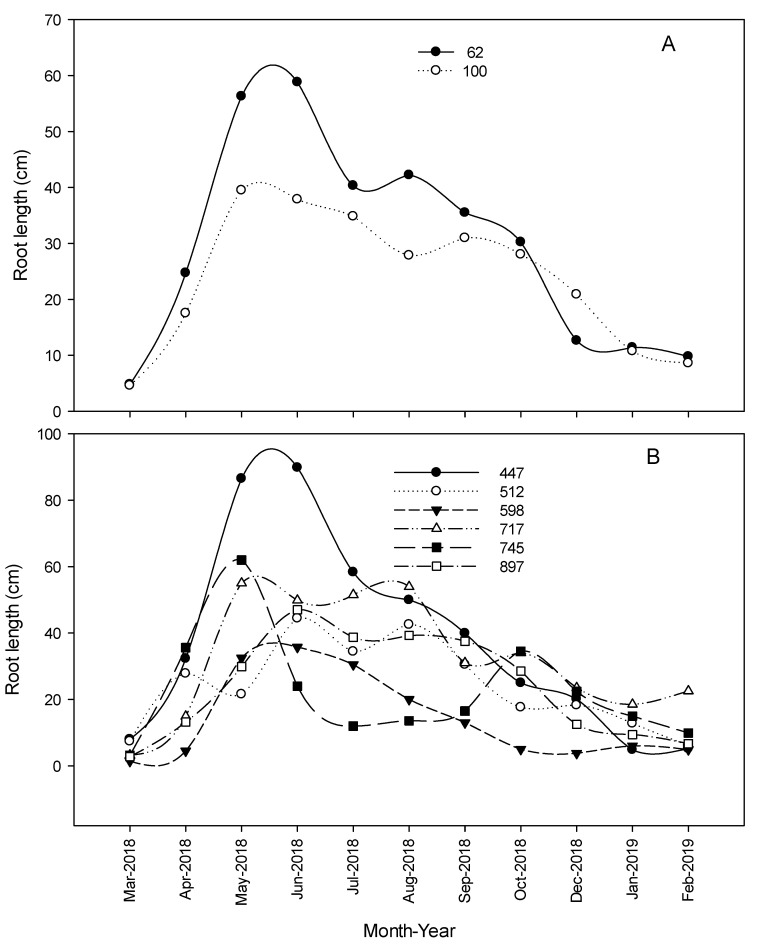
Impact of irrigation rate (**A**) and planting densities (**B**) of young citrus trees on root length growth (cm) during 2018–2019 at the southwest Florida research and education center demonstration grove.

**Figure 3:**
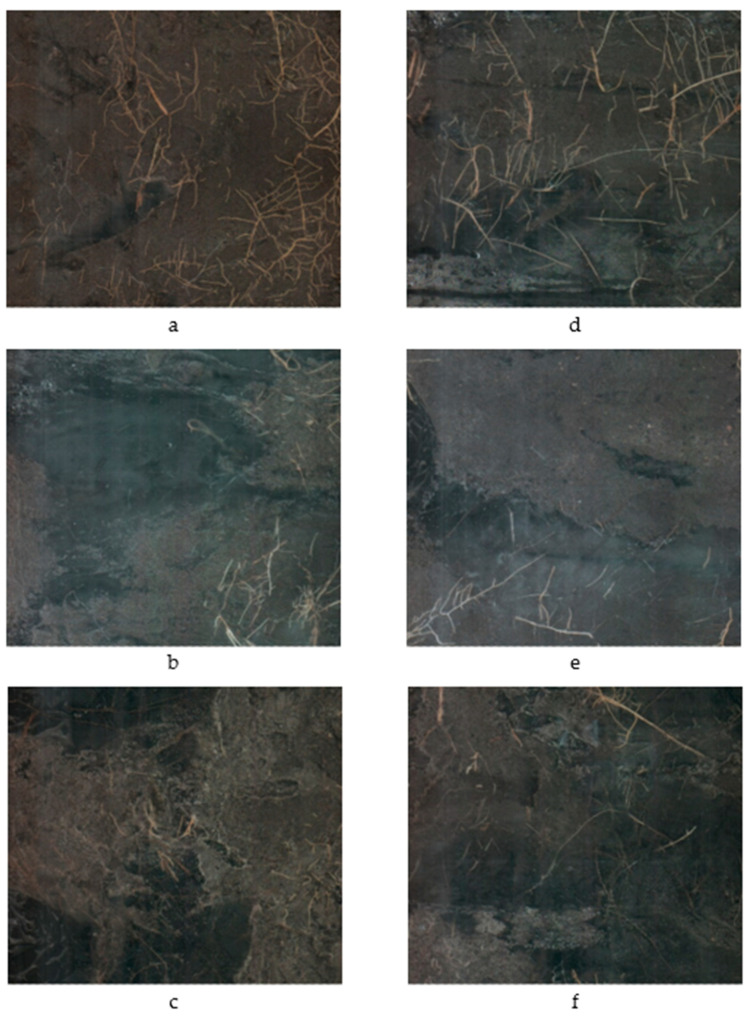
Root images during growing season of 2018 represented the effect of tree densities and irrigation treatments on root development. Images (**a**–**c**) represent 100% ET irrigation rate and images (**d**–**f**) represented 62% ET irrigation rate at 447, 598, and 745 trees per ha, respectively.

**Figure 4:**
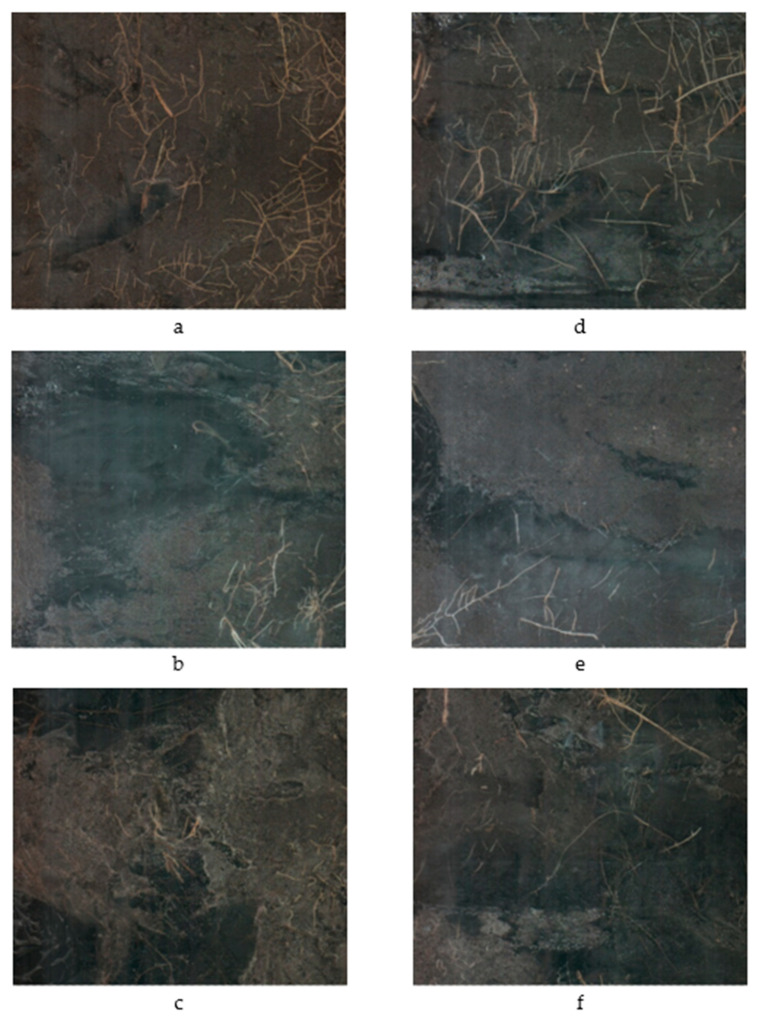
Root images during the growing season of 2018 represented the effect of tree densities and irrigation treatments on root development. Images (**a**–**c**) represent 100% ET irrigation rate and images (**d**–**f**) represented 62% ET irrigation rate at 512, 717, and 897 trees per acre, respectively.

The two-way interaction irrigation × planting density (*p* = 0.0094) significantly impacted the total root length (TRL) of young citrus trees (Figure 2, Figure 3 and Figure 4). Reducing the irrigation from 100% to 62% ETc significantly increased TRL of citrus trees on average by 32% compared with 100% ET_c_ rate (Table 3).

leTrees densities also statistically affected TRL development, with a maximum increase at 447 trees per ha. Based on the number of trees per hectare, lower planting density (447 trees per ha) received less water than the rest of planting densities (each tree was irrigated with either 62% or 100% ETc regardless of planting density during the first 15 months). Such condition enhanced trees strategy to avoid low water potential [23] by relying on mechanisms that maintain the plant’s water status balance, such as restricting shoot growth, leads to an increased root/shoot ratio [24]. Maximum TRL was observed under lower irrigation rate during the summer of 2018 and was 98, 85, 79, 72, 68, and 49 cm under 447, 717, 745, 512, 897, and 598 trees per ha, respectively with an average increase by 40, 62, 18, 49, and 37%, respectively. However, the average daily TRL increase was estimated to be 9.9 and 7.9 mm d^−1^ under 62% and 100% ET_c_, respectively. These results were similar to those published by Bevington and Castle [25] and Hamido et al. [6] where TRL of orange trees increased by almost 10 or 4 mm d^−1^ during the warmer season. Differences could be related to different citrus cultivar and management practices.

The two-way interaction irrigation × planting density (*p* = 0.0328) significantly impacted the number of roots per live image (NOR) developed during measurements. Lower irrigation rate significantly increased NOR of citrus trees on average by 29% compared with 100% rate. Maximum NOR was observed under the lower irrigation rate during the summer of 2018 and was 17, 9, 10, 17, 15, and 10 under 447, 512, 598, 717, 745, and 897 trees per ha, respectively.

**Figure 5:**
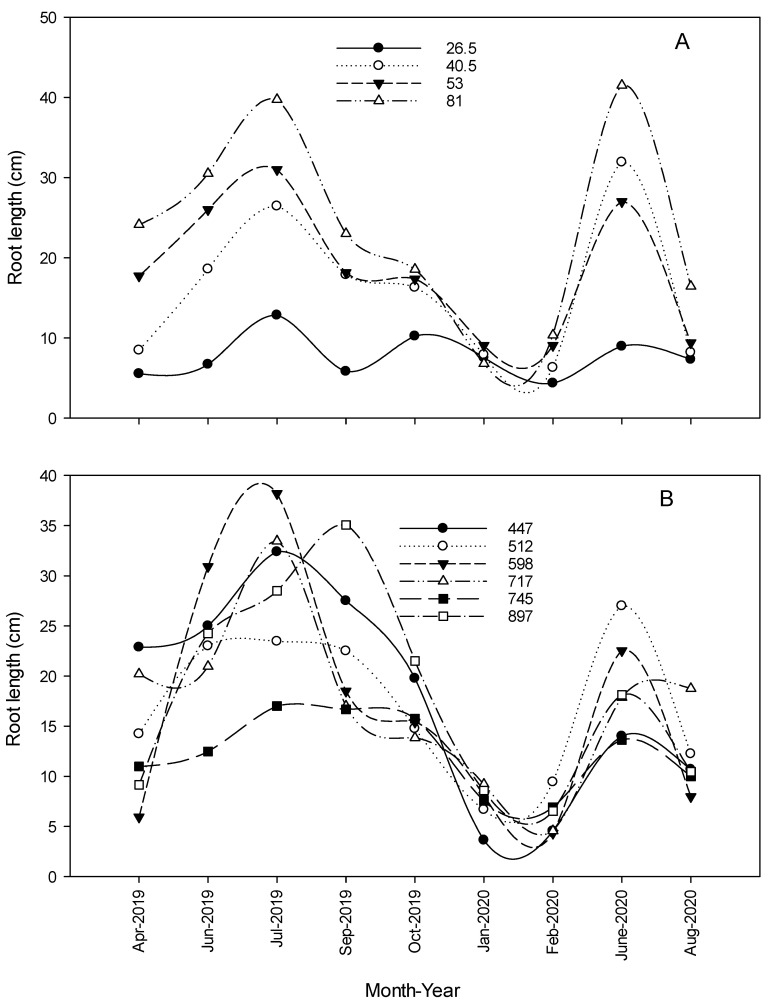
Impact of irrigation rate (**A**) and planting densities (**B**) of young citrus trees on root length growth (cm) during 2018–2019 at the southwest Florida research and education center demonstration grove.

**Figure 6:**
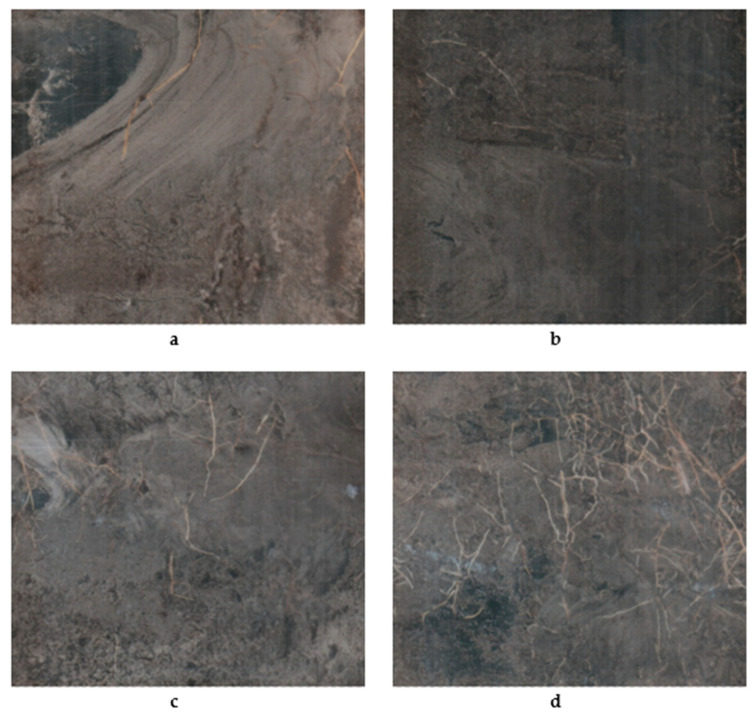
Impact of irrigation rates of 26.5% (**a**), 40.5% (**b**), 53% (**c**), and 81% (**d**) ETo on young citrus trees root growth during growing season in 2019 at the southwest Florida research and education center demonstration grove.

**Figure 7:**
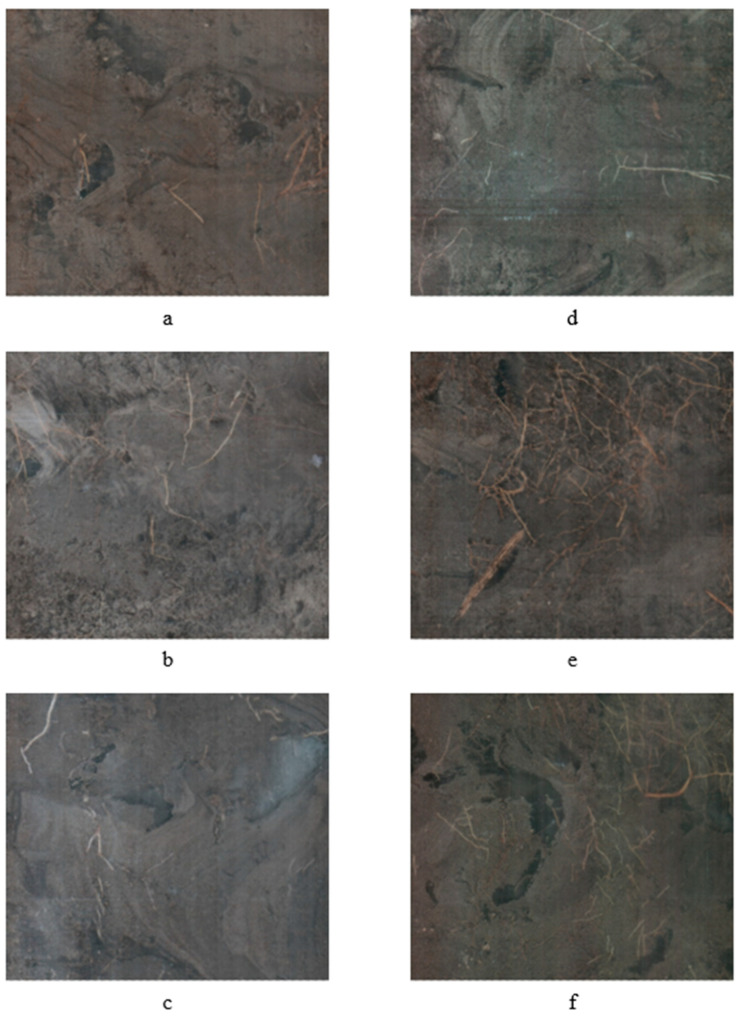
Impact of planting densities of 447 (**a**), 512 (**b**), 598 (**c**), 717 (**d**), 745 (**e**), and 897 (**f**) young citrus trees per hectare on root growth during growing season in 2019 at the southwest Florida research and education center demonstration grove.

When treatments were adjusted, from April 2019 through September 2020, the irrigation rate alone (*p* = 0.0014) significantly impacted the TRL of young citrus trees. Increasing the irrigation by 54.5% significantly increased TRL of citrus trees on average by ≈ twofold (Table 4 and Figure 5, Figure 6 and Figure 7). A similar conclusion was drawn by Alves et al. [21], they assumed the reason could be related to the wetted area under a higher irrigation rate.

However, that was not the case here; all trees were irrigated with the same pump and wetted the same area at different rates; thus, the reason could be related to the length of time that the same surface stayed wet. Thus, water availability determines distribution and length of roots [26].

Maximum TRL was observed during the summer of 2019 and was 17, 52, 43, and 86 cm under 26.5%, 40.5%, 53%, and 81% ET_c_ irrigation rate, respectively. The average daily increase of TRL growth increased from 2.4 to 5.5 mm d^−1^ under 26.5 and 81% ET_c_ rate, respectively.

The two-way interaction irrigation × planting density (*p* = 0.0158) significantly enhanced NOR found during measurements. Increasing the irrigation rate by 54.5% ET_c_ significantly increased NOR of citrus trees on average more than twofold. Maximum NOR was observed under different irrigation rates during the summer of 2019 and was 6, 7, 10, and 14 roots under 26.5%, 40.5%, 53%, and 81% ET_c_ irrigation rate, respectively.

#### 3.1.6. Average Root Diameter

In 2018, the two-way interaction irrigation × planting density (*p* = 0.0100) significantly impacted the average root diameter (ARD) during measurements. However, the irrigation rate did not significantly (*p* = 0.8065) affect the average root diameter. Measured ARD values ranged between 4.3 and 7.3 mm under 512 and 598 trees per ha, respectively.

When treatments were adjusted from April 2019 through September 2020, the two-way interaction irrigation × planting density (*p* = 0.457) and planting density alone (*p* = 0.1096) did not significantly affect the ARD changes. However, the irrigation rate significantly (*p* = 0.0182) affected the ARD, increasing the irrigation rate by 54.5% ET_c_ from 26.5% to 81% significantly reduced the ARD by 22%. Trees produced thicker feeder roots when exposed to water stress [27]. Under lower water availability, plant growth could be reduced, and the growth of roots preferred over leaves.

#### 3.1.7. Root Longevity

**Figure 8:**
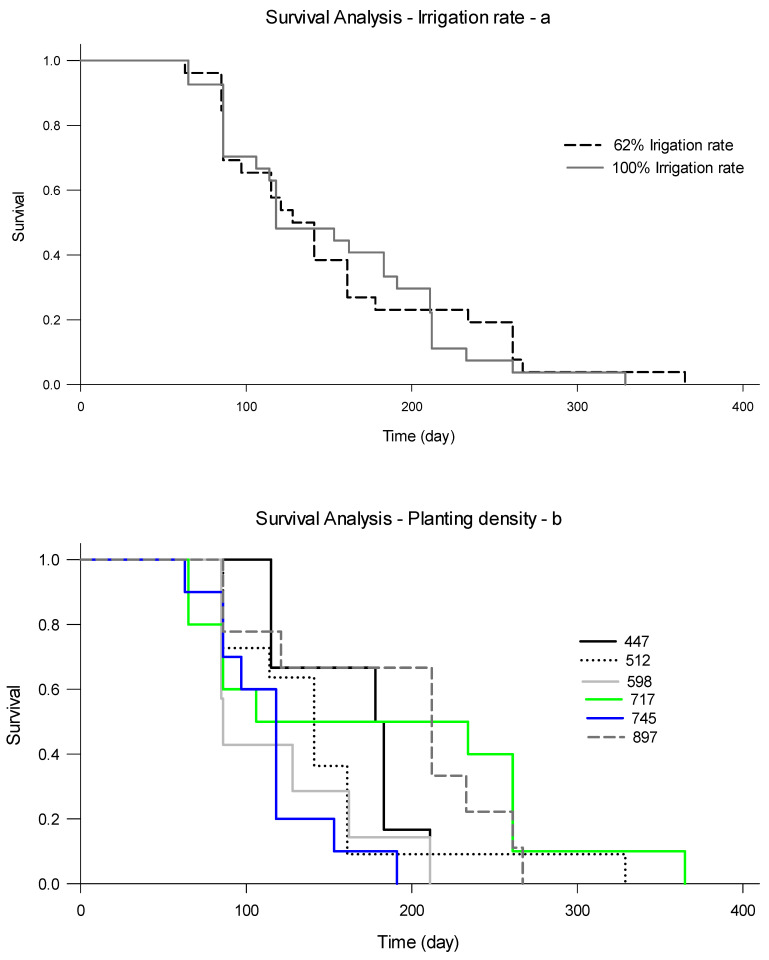
Impact of irrigation rate (**a**) and planting densities (**b**) of young citrus trees on root survival during 2018–2019 at the southwest Florida research and education center demonstration grove.

Although the two-way interaction irrigation × planting density (*p* < 0.0001) and the planting density alone significantly (*p* = 0.0071) impacted root longevity, irrigation rate did not represent a significant effect (*p* = 0.8471) on root lifespan. The cumulative survival curves of the lower and higher irrigation rates were similar (Figure 8a), with a slightly longer root lifespan (≈2%) in the lower irrigation rate (Table 5). Average lifespan was estimated to be 153 and 150 days for 62% and 100 ET_c_ irrigation rates, respectively. That was supported by the Kaplan–Meier survival analysis estimate of the number of dead roots and root survival probability under both irrigation treatments, indicating that the lower irrigation rate improved the root longevity of young citrus trees. However, planting density significantly impacted the root’s lifespan (Figure 8b and Table 6). The average root lifespan ranged between 115 and 188 days under 598 and 897 trees per ha, respectively, indicating that planting young trees much closer to each other enhanced root’s longevity. Current results represented different lifespan than that reported by [5,28], where they estimated root lifespan for citrus trees ‘Swingle citrumelo’ planted in Florida sandy soils to be between 99 and 169 days, respectively. The reason for longer root lifespan during the current project could be related to different treatment, environmental conditions, physiological status of the trees, and grove management. An earlier study indicated that large differences in root longevity could be closely associated with the physiological state of the tree, and has more influence compared with the surrounding environment on underground processes, and the root responses are indirectly conciliated through the influences of aboveground processes [29]. In addition, McCormmack et al. [30] indicated that root longevity could be significantly influenced by the root concentrations of macronutrients such as carbon, calcium, and nitrogen and the ratios between nitrogen and carbon. Furthermore, calcium concentration is significantly impacted by irrigation rate (data not shown).

**Figure 9:**
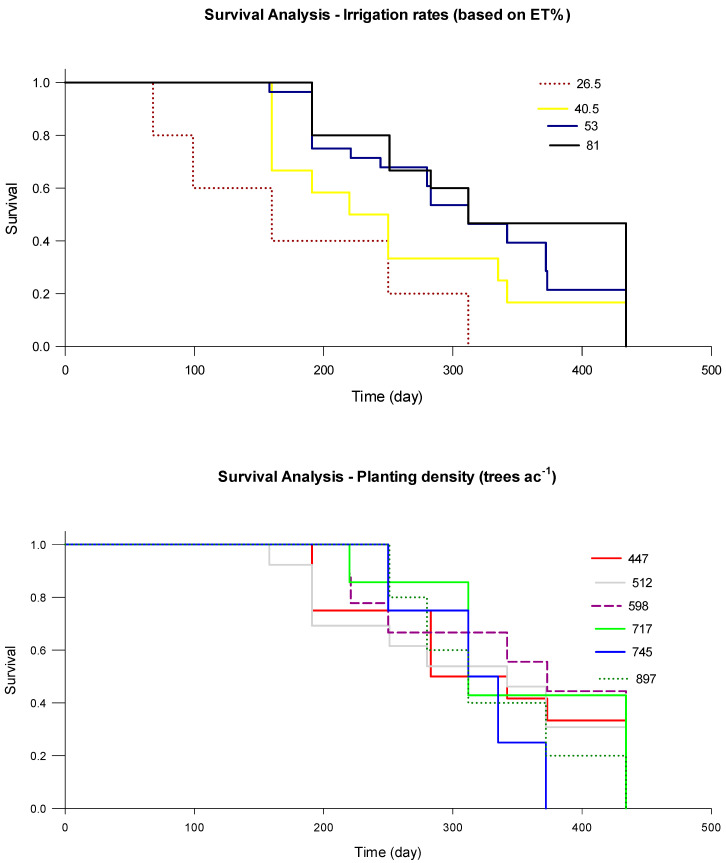
Impact of irrigation rate and planting densities of young citrus trees on root survival during 2019–2020 at the southwest Florida research and education center demonstration grove.

When treatments were adjusted, from April 2019 through September 2020, the two-way interaction irrigation rate × planting density (*p* = 0.3434) and the planting density alone (*p* = 0.2640) did not significantly impact root longevity. However, irrigation rate alone significantly (*p* = 0.0442) affected young citrus tree’s root lifespan. Increasing the irrigation rate to 81% ET_c_ apparently increased soil moisture contents and insulated the root system against unexpected changes in soil temperature [31]. The cumulative survival curves of the different irrigation rates and planting densities represented some similarity with a significant difference exerted in the percent of roots survived under specific treatments (Figure 9a,b and Table 7). That was supported by the Kaplan–Meier survival analysis estimate of the number of dead roots and root survival probability under different irrigation treatments (Table 8), indicating that the 81% ET_c_ irrigation rate improved the root longevity of young citrus trees. The average root lifespan increased from 178 days to 331 days under 26.5% and 81% ET_c_ irrigation rate, respectively, indicating that increasing the irrigation rate by 54.5% of ET_c_ for young trees enhanced roots longevity. These results were similar to those reported by Atta et al. [32] under field settings, where they found that root lifespan for sweet citrus trees planted in the same soils with different nutrition practices can be between 220–339 days.

## 4. Conclusions

Numerous studies have reported that HLB significantly impacted tree’s feeder roots length, lifespan, and leaf area. As a result, the reduction in growth and yield of HLB affected trees. Thus, citrus growers have been forced to plant more trees per hectare to compensate for the losses from HLB. However, if growers plant more trees per unit surface area would they require more water? Current research indicates that the response of citrus trees to irrigation rates varies substantially. In the first year, a lower irrigation rate (62% ETc) exerted promising results during the watered period, which would be favorably applied in commercial citrus groves. The 62% ET_c_ irrigation treatment promoted citrus trees’ root and shoot development. However, during the second and third years, increasing the irrigation rate to 81% ET_c_ significantly enhanced the tree growth, including the tree’s height, trunk diameter, canopy volume, root growth, and root lifespan. Besides, plant growth at higher irrigation rate improved as a result of the adjustment of the plant microenvironment. Thus, the application of 81% ETc irrigation rate in citrus groves is more cost-effective than the traditional full treatments under the southwest Florida conditions. Therefore, the reliable information presented in this work on improved irrigation management of high density citrus plantings could be disseminated to growers for improving tree growth and development under commercial applications.

## Figures and Tables

**Table 1:** Average tree height, trunk diameter (TD), leaf area (LA), and canopy volume (CV) measurements of the young citrus trees under different irrigation rates (I) at different density (D) with dependent variables measured over time at the demonstration grove at the Southwest Florida Research and Education Center.

Model Variables and Interaction	February 2018		May 2018		December 2018				Differences in Growth *		
	Height	TD	TD	LA	Height	TD	LA	CV	Height	TD	LA
	*p* > F **										
I	0.2156	0.1104	0.7513	0.0061	0.5323	0.0306	0.3300	0.1564	0.6688	0.0527	0.6341
D	0.1414	0.9938	0.8062	0.9094	0.0034	0.5044	<0.0001	0.3888	0.0094	0.5058	0.0029
I × D	0.1524	0.1550	0.4120	0.9197	0.0468	0.8716	0.9303	0.8376	0.1983	0.8494	0.9200
**Main effect means**											
**Irrigation rate**	cm	mm	mm	m^2^	cm	mm	m^2^	m^3^			
62%	74.2	6.93	9.86	0.27	116	19.5	1.96	0.2430	41.8	12.7	1.69
100%	74.9	7.02	9.81	0.16	115	20.1	1.89	0.2218	40.1	13.1	1.73
**D (trees per ha)**											
447	74.9	6.98	9.77	0.25	115	19.2	1.44	0.2332	40.1	12.2	1.19
512	74.4	6.96	9.91	0.23	106	20.3	1.83	0.1993	31.6	13.3	1.60
598	72.4	6.96	9.60	0.23	118	19.2	1.74	0.2418	45.6	12.2	1.51
717	74.9	6.98	9.75	0.20	120	19.8	1.99	0.2324	45.1	12.8	1.79
745	73.6	6.97	9.88	0.20	124	19.9	1.78	0.2548	50.4	12.9	1.58
897	75.8	6.99	9.96	0.19	114	20.2	2.50	0.2443	38.2	13.2	2.31

* Differences in growth were estimated from the differences between the first and last measurement for the same parameter. ** *p* Values were obtained from Statistical Analysis System (SAS) analysis.

**Table 2:** Average tree height, trunk diameter (TD), and canopy volume (CV) measurements of the young citrus trees under different irrigation rates (I) at different density (D) with dependent variables measured over time at the demonstration grove at the Southwest Florida Research and Education Center.

Model Variables and Interaction	May 2019			December 2019			June 2020			Differences in Growth		
	Height	TD	CV	Height	TD	CV	Height	TD	CV	Height	TD	CV
	*p* > F											
I	0.0004	0.0063	0.0142	0.0049	0.0026	<0.0001	<0.0001	0.0001	<0.0001	0.0242	0.0282	<0.0001
D	0.1186	0.0124	0.3322	0.1388	0.1655	0.0062	0.6257	0.6410	0.0074	0.4487	0.6195	0.0186
I × D	0.9386	0.2953	0.5278	0.6819	0.3197	<0.0001	0.0459	0.2297	<0.0001	0.0600	0.0577	<0.0001
**Main effect means**												
I	cm	mm	m^3^	cm	mm	m^3^	cm	mm	m^3^	cm	mm	m^3^
26.5	120	22.0	0.48	129	31.7	1.06	128	34.5	1.30	8	12.5	0.82
40.5	122	23.6	0.53	130	32.1	1.56	131	35.6	1.82	9	12	1.29
53	113	22.6	0.57	137	35.3	1.72	139	37.4	2.27	26	14.8	1.70
81	116	23.8	0.74	140	34.1	1.81	150	39.9	3.11	34	16.1	2.37
**D (trees per ha)**												
447	115	21.3	0.55	135	33.4	1.61	138	38.0	2.61	23	16.7	2.06
512	113	22.7	0.42	124	29.8	1.34	135	35.1	2.20	22	12.4	1.78
598	119	21.9	0.54	138	34.8	1.84	140	38.6	2.59	21	16.7	2.05
717	112	23.3	0.48	127	31.6	1.27	133	36.7	2.15	21	13.4	1.67
745	122	23.4	0.72	139	34.6	1.86	143	38.3	2.52	21	14.9	1.80
897	116	24.2	0.53	131	32.4	1.51	135	36.2	2.25	19	12.0	1.72

**Table 3:** Effect of irrigation rates (I) and different planting density (D) on root development and lifespan of citrus tree roots during 2018 and 2019 measurements at the demonstration grove at the southwest Florida research and education center.

Model Variables and Interaction	Number of Roots *	Root Length (cm) *	Average Root Diameter (mm)	Root Lifespan (days) ¶
	*p* > F			
I	<0.0001	<0.0001	0.7692	0.8471
D	0.0002	<0.0001	0.0186	0.0071
M	<0.0001	<0.0001	0.0020	-
I × D	<0.0001	<0.0001	0.0044	˂0.0001
I × D × M	0.0053	0.0005	0.3088	-
**Main effect means**				
I				
62%	9	32.3	6.1	153
100%	7	24.5	6.0	150
**D (trees per ha)**				
447	10	43.1	6.6	164
512	8	26.1	4.3	146
598	6	16.5	7.3	120
717	9	35.5	5.1	179
745	6	22.2	6.8	115
897	8	27.1	6.4	188

* represents the average number of roots that was calculated from monthly measurements between 2018 and 2019. **¶** represents the average lifespan for three or five roots per tree under each planting density.

**Table 4:** Effect of irrigation rates (I), different planting density (D), and the month of measurements (M) on root development and lifespan of citrus tree roots during 2019 and 2020 measurements at the demonstration grove at the southwest Florida research and education center.

Model Variables and Interaction	Number of Roots *	Root Length (cm)*	Average Root Diameter (mm)	Root Lifespan (days) ¶
	*p* > F			
I	0.0018	0.0198	0.0207	0.0442
D	0.4637	0.5616	0.1337	0.2640
M	<0.0001	<0.0001	0.0226	-
I × D	0.0040	0.0873	0.0312	0.3434
I × D × M	0.9944	0.9987	0.4794	-
**Main effect means**				
I				
26.5	2.4	7.8	0.90	178
40.5	4.0	11.6	0.81	258
53	4.1	10.7	0.74	305
81	4.9	14.8	0.71	331
**D (trees per ha)**				
447	4.6	12.5	0.86	296
512	4.7	12.1	0.66	294
598	4.1	12.0	0.79	339
717	4.7	14.1	0.76	329
745	2.8	8.2	0.79	199
897	4.1	12.2	0.82	298

* represents the average number of roots that was calculated from monthly measurements between 2019 and 2020. ¶ represents the average lifespan for three or five roots per tree under each planting density.

**Table 5:** Results of the Kaplan–Meier estimate of root survival under different irrigation rates during 2018 and 2019 measurements at the demonstration grove at the southwest Florida research and education center.

Time of Event (Days)		Number of Roots Died		Live at the Start of the Day (n)		Survival Probability		Standard Error	
62% ETc Irrigation	100% ETc Irrigation	62% ETc Irrigation	100% ETc Irrigation	62% ETc Irrigation	100% ETc Irrigation	62% ETc Irrigation	100% ETc Irrigation	62% ETc Irrigation	100% ETc Irrigation
63	65	1	2	26	27	0.962	0.926	0.0377	0.0504
85	86	3	6	25	25	0.846	0.704	0.0708	0.0879
86	106	4	1	22	19	0.692	0.667	0.0905	0.0907
97	114	1	1	18	18	0.654	0.63	0.0933	0.0929
115	118	2	4	17	17	0.577	0.481	0.0969	0.0962
121	153	1	1	15	13	0.538	0.444	0.0978	0.0956
128	162	1	1	14	12	0.5	0.407	0.0981	0.0946
141	183	3	2	13	11	0.385	0.333	0.0954	0.0907
161	191	3	1	10	9	0.269	0.296	0.087	0.0879
178	211	1	2	7	8	0.231	0.222	0.0826	0.08
234	212	1	3	6	6	0.192	0.111	0.0773	0.0605
261	233	3	1	5	3	0.0769	0.0741	0.0523	0.0504
267	261	1	1	2	2	0.0385	0.037	0.0377	0.0363
365	329	1	1	1	1	0	0	0	0

**Table 6:** Kaplan–Meier estimate of root survival under different planting density during 2018 and 2019 measurements at the demonstration grove at the southwest Florida research and education. center.

Time of Event (days)	Number of Roots Died	Live at the Start of the Day (n)	Survival Probability	Standard Error
Planting density of 447 trees per ha				
115	2	6	0.667	0.192
178	1	4	0.5	0.204
183	2	3	0.167	0.152
211	1	1	0	0
Planting density of 512 trees per ha				
86	3	11	0.727	0.134
114	1	8	0.636	0.145
141	3	7	0.364	0.145
161	3	4	0.0909	0.0867
329	1	1	0	0
Planting density of 598 trees per ha				
85	3	7	0.571	0.187
86	1	4	0.429	0.187
128	1	3	0.286	0.171
162	1	2	0.143	0.132
211	1	1	0	0
Planting density of 717 trees per ha				
65	2	10	0.8	0.126
86	2	8	0.6	0.155
106	1	6	0.5	0.158
234	1	5	0.4	0.155
261	3	4	0.1	0.0949
365	1	1	0	0
Planting density of 745 trees per ha				
63	1	10	0.9	0.0949
86	2	9	0.7	0.145
97	1	7	0.6	0.155
118	4	6	0.2	0.126
153	1	2	0.1	0.0949
191	1	1	0	0
Planting density of 897 trees per ha				
86	2	9	0.778	0.139
121	1	7	0.667	0.157
212	3	6	0.333	0.157
233	1	3	0.222	0.139
261	1	2	0.111	0.105
267	1	1	0	0

**Table 7:** Results of the Kaplan–Meier estimate of root survival under different irrigation rates during 2019 and 2020 measurements at the demonstration grove at the southwest Florida research and education center.

Time of Event (days)				Number of Roots Died				Live at the Day (n)				Survival Probability				Standard Error			
26.5% ETc	40.5% ETc	53% ETc	81% ETc	26.5% ETc	40.5% ETc	53% ETc	81% ETc	26.5% ETc	40.5% ETc	53% ETc	81% ETc	26.5% ETc	40.5% ETc	53% ETc	81% ETc	26.5% ETc	40.5% ETc	53% ETc	81% ETc
68	160	158	191	1	4	1	3	5	12	28	15	0.80	0.67	0.96	0.80	0.18	0.14	0.04	0.10
99	191	191	251	1	1	6	2	4	8	27	12	0.60	0.58	0.75	0.67	0.22	0.14	0.08	0.12
160	220	221	283	1	1	1	1	3	7	21	10	0.40	0.50	0.71	0.60	0.22	0.14	0.09	0.13
250	250	244	312	1	2	1	2	2	6	20	9	0.20	0.33	0.68	0.47	0.18	0.14	0.09	0.13
312	335	280	434	1	1	2	7	1	4	19	7	0.00	0.25	0.61	0.00	0.00	0.13	0.09	0.00
	342	283			1	2			3	17			0.17	0.54			0.11	0.09	
	434	312			2	2			2	15			0.00	0.46			0.00	0.09	
		342				2				13				0.39				0.09	
		372				3				11				0.29				0.09	
		373				2				8				0.21				0.08	
		434				6				6				0.00				0.00	

**Table 8:** Kaplan–Meier estimate of root survival under different planting density during 2019 and 2020 measurements at the demonstration grove at the southwest Florida research and education center.

Time of Event (days)	Number of Roots Died	Live at the Start of the day (n)	Survival Probability	Standard Error
Planting density of 447 trees per ha				
191	3	12	0.75	0.13
283	3	9	0.50	0.14
342	1	6	0.42	0.14
373	1	5	0.33	0.14
434	4	4	0.00	0.00
Planting density of 512 trees per ha				
158	1	13	0.92	0.07
19	3	12	0.69	0.13
251	1	9	0.62	0.14
280	1	8	0.54	0.14
342	1	7	0.46	0.14
372	2	6	0.31	0.13
434	4	4	0.00	0.00
Planting density of 598 trees per ha				
220	1	9	0.89	0.11
221	1	8	0.78	0.14
250	1	7	0.67	0.16
342	1	6	0.56	0.17
373	1	5	0.44	0.17
434	4	4	0.00	0.00
Planting density of 717 trees per ha				
220	1	7	0.86	0.13
312	3	6	0.43	0.19
434	3	3	0.00	0.00
Planting density of 745 trees per ha				
250	1	4	0.75	0.22
312	1	3	0.50	0.25
335	1	2	0.25	0.22
372	1	1	0.00	0.00
Planting density of 897 trees per ha				
251	1	5	0.80	0.18
280	1	4	0.60	0.22
312	1	3	0.40	0.22
372	1	2	0.20	0.18
434	1	1	0.00	0.00
